# HIV Incidence and Predictors of Incident HIV among Men Who Have Sex with Men Attending a Sexual Health Clinic in Melbourne, Australia

**DOI:** 10.1371/journal.pone.0156160

**Published:** 2016-05-24

**Authors:** King T. Cheung, Christopher K. Fairley, Tim R. H. Read, Ian Denham, Glenda Fehler, Catriona S. Bradshaw, Marcus Y. Chen, Eric P. F. Chow

**Affiliations:** 1 Melbourne Sexual Health Centre, Alfred Health, Melbourne, VIC, Australia; 2 Melbourne Medical School, Faculty of Medicine, Dentistry and Health Sciences, University of Melbourne, Melbourne, VIC, Australia; 3 Central Clinical School, Faculty of Medicine, Nursing and Health Sciences, Monash University, Melbourne, VIC, Australia; Fudan University, CHINA

## Abstract

**Introduction:**

The aim of this study was to determine the risk factors for HIV infection and the incidence in men who have sex with men (MSM). It is important to identify subgroups of MSM in which preventive interventions such as pre-exposure prophylaxis (PrEP) offered at the time of their last negative test would be considered cost-effective.

**Methods:**

We conducted a retrospective cohort study of MSM attending Melbourne Sexual Health Centre (MSHC) during 2007–2013 with at least two HIV tests within 12 months of each other. Demographic characteristics, sexual and other behaviours, and bacterial sexually transmitted infection (STI) diagnoses were extracted from the date of the last negative HIV test. HIV incidence rate (IR) per 100 person-years for each risk factor was calculated.

**Results:**

Of the 13907 MSM who attended MSHC, 5256 MSM had at least two HIV tests and were eligible, contributing 6391 person-years follow-up. 81 new HIV diagnoses were identified within 12 months of an HIV negative test with an incidence of 1.3 (95% CI: 1.0–1.6) per 100 person-years. Significant associations with subsequent HIV infection were: rectal gonorrhea (HIV IR: 3.4 95% CI: 2.1–5.2), rectal chlamydia (HIV IR: 2.6 95% CI: 1.7–3.7), inconsistent condom use (HIV IR: 2.1 95% CI: 1.6–2.7), use of post-exposure prophylaxis (HIV IR: 2.3 95% CI: 1.7–3.1), and injecting drug use (HIV IR: 8.5 95% CI: 3.4–17.5).

**Conclusion:**

The incidence of HIV was above 2.0% in subgroups of MSM with specific characteristics at the last HIV negative test. PrEP is considered cost effective at this incidence and could potentially be used along with other preventive interventions for these individuals in more than half of the population.

## Introduction

Pre-exposure prophylaxis for HIV (PrEP) is expensive; however, it becomes more cost-effective when offered to populations with a higher incidence of HIV infection, particularly when it is above 2% per year. [1–3] It may be that using risk factors to identify subgroups with a higher incidence of HIV will allow PrEP to be used in a cost-effective way.

Studies in the literature often focus on risk factors reported by individuals at or after their HIV diagnosis. [4–11] A large cohort study in Italy demonstrated a link between previous syphilis diagnosis and HIV infection among MSM. [10] In Australia, a community-based cohort study in Sydney has showed rectal gonorrhea and anal warts were independent risk factors for HIV acquisition; however, this study did not examine demographic and behavioural characteristics. [12] Risk factors reported by individuals may have been different at the time of the last negative HIV test, when an intervention such as PrEP may have been possible, rather than at the time of diagnosis. In addition, these studies do not quantify the risk of HIV among HIV-negative individuals, and therefore they cannot be used to identify those subgroups of MSM where the risk of HIV is high enough to justify more expensive interventions. Sexual health clinics are an ideal setting to study factors predictive of HIV because the recording of sexual risk data, and HIV/STI testing are all undertaken routinely and repeatedly in MSM.

The aim of this study was to identify risk factors for HIV infection at the time of the last negative HIV test in MSM attending a sexual health centre. We specifically looked at risk factors associated with HIV diagnosis within 12 months of a negative HIV test. In particular, we were interested in whether an STI diagnosis within the past 12 months increased the risk of subsequent incident HIV infection in MSM as this would be a relevant and practical time to offer HIV preventive interventions.

## Methods

### Study population and setting

This was a retrospective cohort study at Melbourne Sexual Health Centre (MSHC), the largest public sexual health clinic in the State of Victoria, Australia. It provides about 35,000 consultations per year, and all services are free. Approximately 37% of these are MSM consultations, and about 30% of MSM attended MSHC with noticeable symptoms. The HIV prevalence among MSM at MSHC is about 1%.[13] The clinic has an electronic medical record system that includes demographic and epidemiological data. We only included MSM who had at least two HIV tests within 12 months, from 1^st^ January 2007 to 31^st^ December 2013. MSM were defined as men who had sex with another man in the last 12 months. Transgender individuals were excluded. Furthermore, MSM who tested positive for HIV on their first visit at MSHC or were known to have HIV at their first consultation were excluded.

### Data collection

Age, previous use of post-exposure prophylaxis (PEP), sexual (i.e. sex outside Australia, number of sexual partners and condom use), and injecting drug use behaviour for eligible MSM were included in the analysis. PEP use data were extracted from the clinical database, and behavioural data were from the 12 months prior to the consultation and were self-reported by patients using computer-assisted self-interviewing. MSHC followed the Australian STI guidelines for MSM screening during the study period [14], which recommended annual screening for pharyngeal gonorrhoea, rectal chlamydia and gonorrhoea, and urethral chlamydia among MSM; screening for pharyngeal chlamydia and urethral gonorrhoea were not recommended for asymptomatic MSM.

Laboratory diagnoses of gonorrhea (pharyngeal, urethral, or rectal), chlamydia (urethral, or rectal), infectious syphilis and HIV were extracted. BD ProbeTec Strand Displacement Amplification Assay (Becton, Dickinson and Company, Sparks, MD, USA) was used for the detection of urethral and rectal chlamydia. [15, 16] Rectal, urethral, and pharyngeal swabs for gonorrhoea were plated onto GC Agar medium for culture. All syphilis cases were detected by using *Treponema pallidum* enzyme immunoassay (EIA) and confirmed by *T*. *pallidum* particle agglutination assay (TPPA), and Rapid Plasma Reagin (RPR) test. HIV infection was determined by third generation enzyme immunoassay (Murex, Dartford, UK) and confirmed by Western Blot.

### Statistical analysis

For each individual the period(s) of observation in the cohort was calculated from an initial HIV negative test to the next HIV test regardless of the result, within a 12-month period. If the individual did not have another HIV test within 12 months of the first HIV test, this period was not included in the observation period. We then repeated the above process for the next HIV test. In this way an individual could have a number of different periods of observation. We excluded periods of observation where no STI testing was performed in the previous 12 months.

The HIV incidence rate was calculated as the total number of new HIV diagnoses divided by the total person-years-at-risk, expressed as cases per 100 person-years. Person-years-at-risk was calculated as the sum of observation periods that had a STI test at baseline or in the past 12 months.

For characteristics associated with an HIV incidence of 2% or more, we calculated the proportion of individuals with the identified characteristics at the initial consultations among all MSM, and this was calculated by dividing the number of consultations with characteristics by the total number of consultations. The population attributable fraction (PAF) for HIV associated with these risk factors was also calculated.

MSM who were diagnosed with any STI (i.e. gonorrhoea, chlamydia or syphilis) within 6 weeks of HIV diagnosis were excluded to reduce the likelihood of simultaneously acquired infections where the HIV test was initially negative due to the time required to seroconvert. Incidence rate (IR) and rate ratio (RR) for HIV were calculated for each risk factor. Rate ratios were computed as the ratio of the incidence rate in the category of interest divided by the rate in the referent category for each risk factor, and the 95% confidence intervals of IR and RR were calculated based on exact Poisson methods. All statistical analyses were performed using Intercooled Stata 13.1 (Stata Corp, College Station, TX, USA).

### Ethical statement

Ethical approval was obtained from the Ethics Committee of Alfred Hospital, Melbourne, Australia (number 504/13). No consent was given to the participants. Participant's records and information were anonymised and de-identified prior to analysis.

## Results

There were 13907 individual MSM who attended MSHC over the seven year period, during which 310 were diagnosed with HIV. A total of 5256 of them had at least two HIV tests done within a 12 month period and were HIV negative on the first test. Among these 5256 MSM, 49283 consultations were made, with a total number of 1465 gonorrhea, 1885 chlamydia, and 394 syphilis diagnoses. Eighty-one HIV cases were diagnosed among these 5256 MSM within 12 months of an initial negative HIV test, yielding an HIV incidence of 1.3/100 person-years (95% CI: 1.0–1.6) during the study period.

Diagnosis of rectal gonorrhea in the past 12 months had the strongest association with subsequent HIV acquisition (RR: 3.1; 95% CI: 1.8–5.2) compared to other STIs. Rectal chlamydia diagnosis in the past 12 months increased the risk of HIV infection more than 2-fold (RR: 2.4; 95% CI: 1.5–3.9). MSM were 2.3 (95% CI: 1.4–3.7) and 2.3 (95% CI: 1.4–3.7) times more likely to acquire HIV, respectively, if they had been diagnosed with gonorrhea and chlamydia at any site in the past 12 months. Inconsistent condom use during anal sex (RR: 3.1; 95% CI: 1.8–5.4) and injecting drug use (RR: 7.4; 95% CI: 2.9–16.1) in the last 12 months were associated with the diagnosis of incident HIV in the subsequent 12 months (Table 1). For all significant associations with STIs by site, we repeated the analysis excluding any cases with more than one STI; the association for each was essentially the same except for urethral chlamydia (S1 Table).

**Table 1:** Association between incident HIV infection and demographic characteristics, sexual behaviours and STI diagnosis in the last 12 months.

^^^ Characteristics (in the past 12 months)	No. of HIV diagnoses	Person Years	HIV incidence rate per 100 person-years [95% CI]	Rate Ratio [95% CI]	*p* value
**All**	81	6391	1.3 [1.0, 1.6]	-	-
**Age at consultation**					
< 25	20	1514	1.3 [0.8, 2.0]	1.2 [0.6, 2.4]	0.24
25–29	25	1655	1.5 [1.0, 2.2]	1.4 [0.8, 2.6]	0.12
30–34	13	1070	1.2 [0.6, 2.1]	1.1 [0.5, 2.3]	0.35
≥35	23	2153	1.1 [0.7, 1.6]	1.0 (referent)	-
**Number of male partners**					
<4	14	1374	1.0 [0.6, 1.7]	1.0 (referent)	-
≥ 4	56	4170	1.3 [1.0, 1.7]	1.3 [0.7, 2.6]	0.18
**Condom use during anal sex**					
Consistent	20	2991	0.7 [0.4, 1.0]	1.0 (referent)	-
Inconsistent	60	2912	2.1 [1.6, 2.7]	3.1 [1.8, 5.4]	**< 0.001**
**Had sex with female**					
No	72	5603	1.3 [1.0, 1.6]	1.0 (referent)	-
Yes	9	720	1.2 [0.6, 2.4]	1.0 [0.4, 2.0]	0.49
**Injecting drug use**					
No	64	5565	1.2 [0.9, 1.5]	1.0 (referent)	-
Yes	7	83	8.5 [3.4, 17.5]	7.4 [2.9, 16.1]	**< 0. 001**
**Sex overseas**					
No	63	4638	1.4 [1.0, 1.7]	1.0 (referent)	-
Yes	16	1500	1.1 [0.6, 1.7]	0.8 [0.4, 1.4]	0.20
**PEP use**					
No	33	4329	0.8 [0.5, 1.1]	1.0 (referent)	-
Yes	48	2062	2.3 [1.7, 3.1]	3.1 [1.9, 4.9]	**< 0. 001**
Any STI diagnosis*					
No	37	4390	0.8 [0.6, 1.2]	1.0 (referent)	
Yes	44	2000	2.2 [1.6, 3.0]	2.6 [1.7, 4.2]	**< 0.001**
**Gonorrhea**					
Any site					
Negative	54	5242	1.0 [0.8, 1.3]	1.0 (referent)	-
Positive	27	1134	2.4 [1.6, 3.5]	2.3 [1.4, 3.7]	**< 0.001**
Pharyngeal					
Negative	72	5891	1.2 [1.0, 1.6]	1.0 (referent)	-
Positive	8	448	1.8 [0.8, 3.5]	1.5 [0.6, 3.0]	0.16
Urethral					
Negative	16	1471	1.1 [0.6, 1.8]	1.0 (referent)	-
Positive	9	469	1.9 [0.9, 3.6]	1.8 [0.7, 4.2]	0.09
Rectal					
Negative	60	5450	1.1 [0.8, 1.4]	1.0 (referent)	-
Positive	21	614	3.4 [2.1, 5.2]	3.1 [1.8, 5.2]	**< 0.001**
**Chlamydia**					
Any site					
Negative	48	4795	1.0 [0.7, 1.3]	1.0 (referent)	-
Positive	32	1379	2.3 [1.6, 3.3]	2.3 [1.4, 3.7]	**< 0.001**
Urethral					
Negative	41	3566	1.1 [0.8, 1.6]	1.0 (referent)	-
Positive	15	560	2.7 [1.5, 4.4]	2.3 [1.2, 4.3]	**< 0.001**
Rectal					
Negative	53	5010	1.1 [0.8, 1.4]	1.0 (referent)	-
Positive	27	1052	2.6 [1.7, 3.7]	2.4 [1.5, 3.9]	**< 0.001**
**Syphilis**					
Negative	25	2021	1.2 [0.8, 1.8]	1.0 (referent)	-
Positive	7	301	2.3 [0.9, 4.8]	1.9 [0.7, 4.5]	0.08

^^^ Of 81 HIV infected individuals, 11 were missing data on number of male partners; 1 was missing data on condom use; 10 were missing data on injecting drug use; 2 were missing data on sex overseas; Gonorrhoea: 1 and 56 were missing data on pharyngeal and urethral respectively; Chlamydia: 25 and 1 were missing data on urethral and rectal respectively; 49 were missing data on syphilis. PEP = post exposure prophylaxis.

^*^ ‘Any STI diagnosis’ includes gonorrhoea at any sites, chlamydia at any sites, and syphilis.

**Fig 1:**
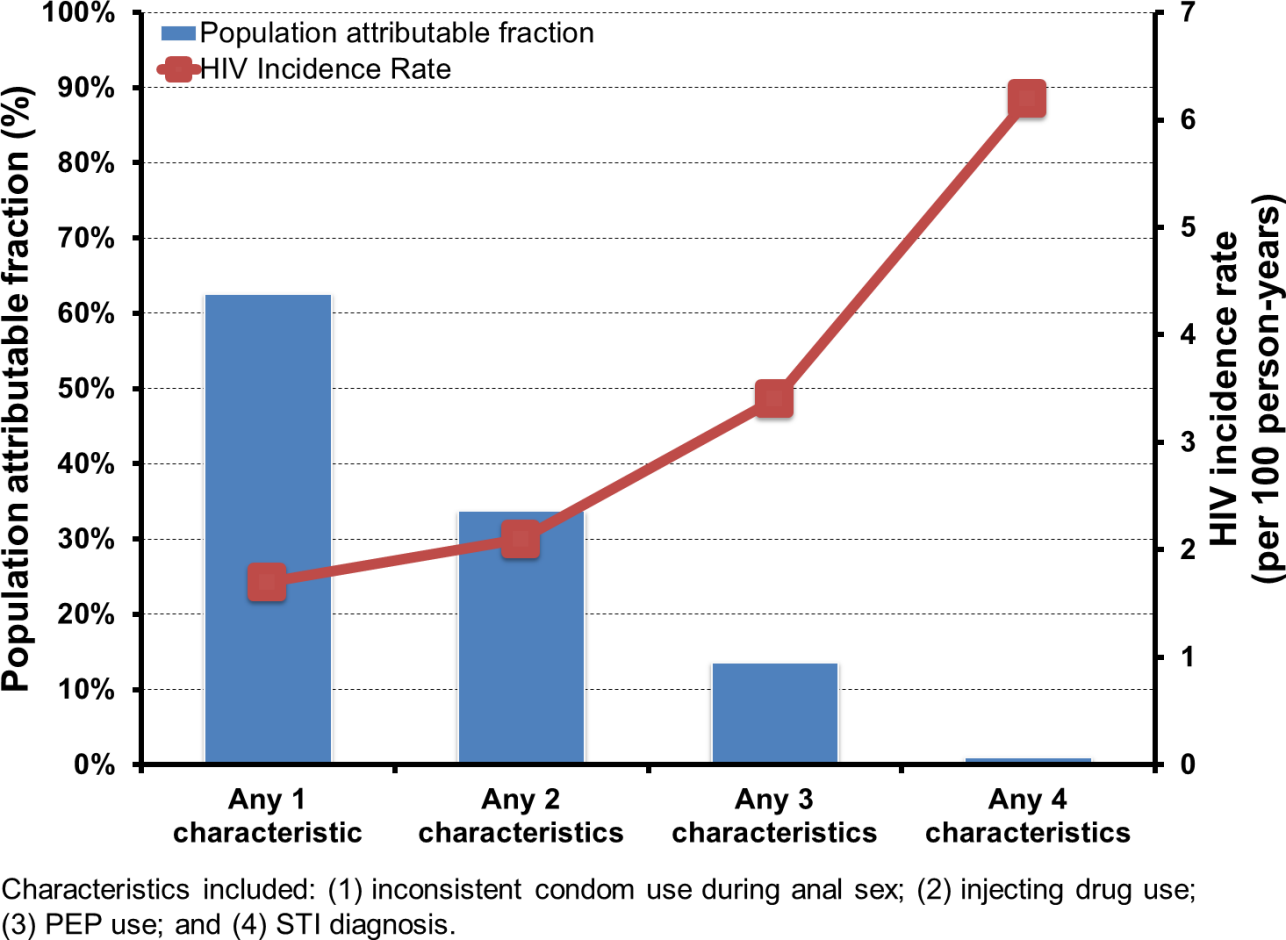
The population attributable fraction for HIV and HIV incidence of different risk characteristics.

Four characteristics (inconsistent condom use during anal sex, injecting drug use, PEP use, and having any STI diagnosis in the last 12 months) were associated with an HIV incidence of 2% or more. Of all MSM included in this study, 52% reported inconsistent condom use, and 37% used PEP in the 12 months before their initial consultations, with HIV incidence rates (IR) of 2.1/100 person-year (95% CI: 1.6–2.7) and 2.3/100 person-year (95% CI: 1.7–3.1), respectively (Table 2). Of the 1.7% who reported injecting drug use in the past 12 months, the observed HIV IR was 8.5 (95% CI: 3.4–17.5). Furthermore, 34% of consultations were preceded by at least one STI diagnosis in the past 12 months and was associated with an HIV IR of 2.2 (95% CI: 1.6, 3.0). The PAF of inconsistent condom use during anal sex was 44.7% (95% CI: 17.5, 62.9), and rectal chlamydia/gonorrhoea infections was 23.9% (95% CI: 5.8, 38.6). Individuals with more than one risk factor were at higher risk of HIV. The proportions of consultations with any one, two, three, or four of these characteristics were 77% (HIV IR: 1.7; 95% CI: 1.3, 2.1), 44% (HIV IR: 2.1; 95% CI: 1.6, 2.8), 10% (HIV IR: 3.4; 95% CI: 1.9, 5.5) and 0.4% (HIV IR: 6.2; 95% CI: 0.2, 34.4) (Fig 1).

**Table 2:** The proportion of consultations, HIV incidence, and population attributable fraction for HIV of particular characteristics.

MSM’s characteristics in the last 12 months	Proportion of those seen at initial consultation with characteristic (%)	HIV incidence rate per 100 person-years[95% CI]	Population attributable fraction (PAF) for HIV (%) [95% CI]
**Inconsistent condom use during anal sex**	52%	2.1 [1.6, 2.7]	44.7 [17.5, 62.9]
**Injecting drug use**	1.7%	8.5 [3.4, 17.5]	7.9 [0.6, 14.6]
**PEP use**	37%	2.3 [1.7, 3.1]	0.8 [0, 18.6]
**STI diagnosis**^**a**^	34%	2.2 [1.6, 3.0]	20.4 [0, 36.6]
Rectal Infections^b^	26%	2.8 [2.0, 3.9]	23.9 [5.8, 38.6]
Non-rectal infections^c^	20%	2.2 [1.5, 3.3]	6.3 [0, 19.9]
Gonorrhoea at any site^d^	20%	2.4 [1.6, 3.5]	5.6 [0, 18.7]
Chlamydia at any site^e^	25%	2.3 [1.6, 3.3]	14.3 [0, 28.4]
**Inconsistent condom use AND rectal gonorrhoea/chlamydia infections**	15%	4.1 [2.8, 5.8]	24.1 [9.5, 36.3]
**Any 1 characteristic**	77%	1.7 [1.3, 2.1]	62.6 [24.1, 81.5]
**Any 2 characteristics**	44%	2.1 [1.6, 2.8]	33.7 [12.0, 50.0]
**Any 3 characteristics**	10%	3.4 [1.9, 5.5]	13.6 [2.1, 23.7]
**Any 4 characteristics**	0.4%	6.2 [0.2, 34.4]	1.0 [0, 3.7]

^a^ Gonorrhoea at any sites, chlamydia at any sites and syphilis

^b^ Rectal gonorrhoea and/or chlamydia

^c^ Pharyngeal and/or urethral gonorhorea, and/or urethral chlamydia

^d^ Pharyngeal, urethral and/or rectal gonorrhoea

^e^ Urethral and/or rectal chlamydia.

## Discussion

We found specific measurable characteristics exist for MSM that indicate they are at high risk of subsequent HIV infection at the time of the last negative HIV test, potentially allowing the targeting of cost-effective PrEP or other expensive interventions in over half of MSM clinic attendees. Rectal infection with gonorrhoea and/or chlamydia in the past 12 months was one of the most important risk factors, predicting an HIV incidence of about 2.8% per year. This was present in one quarter of the entire cohort but predicted almost half of these incident HIV infections, illustrating the potential impact of targeted interventions such as PrEP. [17] Inconsistent condom use during anal sex was associated with an HIV incidence of 2.1% per year, and was reported by half of the cohort. Being aware of these predictors of HIV infection at the time a person is HIV negative could help clinics prioritise those at greatest risk, for interventions such as PrEP, which become cost-effective in those with a higher incidence of HIV.

The strengths of this study include, knowing the HIV-negative status of men entering the cohort, laboratory confirmation of bacterial STI diagnoses rather than self-report, and self-reported behavioural data and other risk factors, all of which were ascertained at a visit preceding the diagnosis of HIV. Several limitations of this study should be considered before applying these predictors to other settings. Some MSM in our cohort would have had HIV or STI diagnoses elsewhere, that we are unaware of, which may bias our results. We excluded about 60% of those initially seen because they had no more than one visit within 12 months during the study period. If the risk factors for HIV in the group we excluded were different to those who were seen again, then our study design was not able to assess this. We also excluded all cases of STI within 6 weeks of HIV diagnosis; however, this 42-day cut-off would also have excluded asymptomatic STI that were present prior to HIV infection, and also included simultaneous infections where the next HIV test was performed more than 42 days after the first test. In Australia, injecting drug use accounts for only about 3% of HIV infections so these findings may not apply to countries where injecting drug use is the primary mode of transmission of HIV. [18] We did not collect data on drugs that were ingested or smoked. Also the data on sexual behaviours are very limited. We could not distinguish the type of anal sex (i.e. receptive or insertive), and therefore were unable to distinguish the risk between the two. Furthermore, the number of sex acts was not collected and hence we were not able to identify the number and nature i.e. insertive or receptive of condomless anal sex acts. Other risk practices such as group sex and anonymous sex are found to be highly associated with HIV and STI but were not collected in this study. Finally, even though MSHC sees a substantial proportion of MSM living in Melbourne, it is possible that the risk factors and incidence estimates are not representative of the broader population of MSM in Victoria. [19]

Our findings are consistent with the findings of other published studies. In 2014, Katz *et al* in Washington showed that previous rectal gonorrhea, among other STIs, was associated with the greatest risk of subsequent HIV acquisition among MSM who had at least one episode of STI, with an incidence of 4.1/100 person-year. [20] Similarly, using data collected at the San Francisco City Clinic, Bernstein *et al* examined the risk of HIV infection in high-risk MSM who had rectal gonorrhea or chlamydia and found that having two additional prior rectal infections was associated with an 8-fold increase in risk (IR: 15.0; 95% CI: [3.2, 37.9]). [21] Furthermore, syphilis is found not to be associated with HIV acquisition, which is consistent with the finding of the Sydney study. [12] Compared to these studies, our analysis was limited to the presence of STI in the past 12 months to focus on the short-term sero-conversion risk among MSM. Among risk factors at HIV diagnosis, self-reported gonorrhea was shown by Koblin in a large cohort study in the United States to be associated with a 4.6 times higher risk of HIV infection in MSM (95% CI: 2.8, 7.6). [22] In addition, chlamydia (HR: 2.2; 95% CI: 1.2, 4.3) and injecting drug use (HR: 2.2; 95% CI: 1.5, 3.3) were also strongly associated with HIV infection. In contrast to our study, MSM with four or more male partners were more likely to acquire HIV in Koblin’s study (HR: 2.8; 95% CI: 1.7, 4.7).

PrEP is considered to be the most expensive preventive intervention available for HIV infection because it involves giving a larger HIV-negative population two-thirds of the medication that would be used to treat HIV. [23] Cost-effectiveness of PrEP depends on multiple factors which will vary in different countries, including the cost of antiretrovirals, the efficacy of PrEP, age and HIV incidence in the population. In one sensitivity analysis, Paltiel *et al*, using a function of these parameters found that an incidence of 2.4%, as compared to 1.6% in their base model, would significantly improve the cost-effectiveness of PrEP. [2] The same improvement was achieved at a lower annual incidence rate if simultaneous changes occurred in the other parameters. This is important because the cost of antiretrovirals is likely to fall as patents on these drugs expire in the near future. On the other hand, using a similar dynamic model with an estimated base incidence of 0.8%, Juusola *et al* also showed that PrEP offered to a population with an annual incidence of 2.3% could be both more effective and cost-effective. [3] The cost-effectiveness of PrEP is also determined by its acceptability to MSM and their awareness of it. Holts in 2013 found that about only 28% of HIV-negative MSM were willing to use PrEP in Australia. [24] In another Australian-based study, the willingness to use PrEP among MSM was associated with younger age and higher number of male sex partners. [25]

In summary, identifying key risk factors for HIV infection enables better direction of prevention efforts at both the individual and population level. MSM with bacterial STI and those reporting inconsistent condom use or PEP use are important targets for HIV prevention because they have the highest incidence and these are relatively common risk factors. Our analysis provides a basis for identifying subgroups with high enough incidence and therefore MSM who report these characteristics should be prioritised for access to PrEP; however, more studies are needed to identify the best combination of risk factors that will identify the largest proportion of MSM at increased risk of HIV infection.

## Supporting Information

S1 Table Association of rectal gonorrhea, urethral and rectal chlamydia with incident HIV infection, excluding cases with more than one STI. (PDF)
