# Supplementary material for: HIV Incidence and Predictors of Incident HIV among Men Who Have Sex with Men Attending a Sexual Health Clinic in Melbourne, Australia
Source: PLoS One. 2016 May 24;11(5):e0156160. doi: 10.1371/journal.pone.0156160 (PMC4878753; doi:10.1371/journal.pone.0156160)
Supplement: S1 Table — (PDF) [file pone.0156160.s001.pdf]

Supplementary Table S1. Association of rectal gonorrhea, urethral and rectal chlamydia with incident HIV infection, excluding cases with more than one STI.

STI in the past 12 months	HIV incidence rate per 100 person-years [95% CI]	Rate Ratio [95% CI]	*p* value
**Rectal gonorrhea**			
Negative	0.9 [0.6, 1.2]	1.0 (referent)	-
Positive	2.8 [0.9, 6.4]	3.1 [1.0, 8.05]	0.018
**Urethral chlamydia**			
Negative	0.8 [0.4, 1.2]	1.0 (referent)	-
Positive	1.0 [0.1, 3.8]	1.4 [0.2, 5.8]	0.310
**Rectal chlamydia**			
Negative	0.9 [0.6, 1.2]	1.0 (referent)	-
Positive	2.0 [1.0, 3.7]	2.3 [1.0, 4.8]	0.014
